# Improving core outcome set development: qualitative interviews with developers provided pointers to inform guidance

**DOI:** 10.1016/j.jclinepi.2017.04.024

**Published:** 2017-06

**Authors:** Elizabeth Gargon, Paula R. Williamson, Bridget Young

**Affiliations:** a Department of Biostatistics, MRC NWHTMR, University of Liverpool, Block F Waterhouse Building, 1-5 Brownlow Street, Liverpool L69 3GL; b Department of Psychology, Health and Society, University of Liverpool, Whelan Building, Brownlow Hill, Liverpool L69 3GB

**Keywords:** Consensus, Core outcome sets, Delphi, Interview, Outcomes, Qualitative research, Research methodology

## Abstract

**Objectives:**

The objective of the study was to explore core outcome set (COS) developers' experiences of their work to inform methodological guidance on COS development and identify areas for future methodological research.

**Study Design and Setting:**

Semistructured, audio-recorded interviews with a purposive sample of 32 COS developers. Analysis of transcribed interviews was informed by the constant comparative method and framework analysis.

**Results:**

Developers found COS development to be challenging, particularly in relation to patient participation and accessing funding. Their accounts raised fundamental questions about the status of COS development and whether it is consultation or research. Developers emphasized how the absence of guidance had affected their work and identified areas where guidance or evidence about COS development would be useful including, patient participation, ethics, international development, and implementation. They particularly wanted guidance on systematic reviews, Delphi, and consensus meetings.

**Conclusion:**

The findings raise important questions about the funding, status, and process of COS development and indicate ways that it could be strengthened. Guidance could help developers to strengthen their work, but over specification could threaten quality in COS development. Guidance should therefore highlight common issues to consider and encourage tailoring of COS development to the context and circumstances of particular COS.

**What is new?**

**Key findings**
• Against a backdrop of limited funding, participants described core outcome set (COS) development as often driven by practicalities rather than principles, and raised fundamental questions about whether COS development is consultation or research.
• Developers problematized the participation of patient stakeholders, particularly their ability to understand COS and prioritize outcomes.
• They did not similarly problematize the participation of professional stakeholders, although they did describe general difficulties in selecting, accessing, and retaining stakeholders.
• Developers also raised questions about whether COS should be developed internationally and aim for generalizability across different countries.
• Finally, developers wanted support on methods of COS development and stakeholder engagement.

**What this adds to what was known?**
• COS development is an emergent area, and little was previously known about developers' experiences and methodological choices.
• As the first inductive study in this area, the findings provide insights about the challenges in developing COS, the areas where developers would benefit from methodological guidance and priorities for future methodological research on COS development.

**What is the implication and what should change now?**
• Limited funding and fundamental uncertainties about whether COS development is consultation or research have wide-ranging implications for COS, and these issues warrant further attention.
• Developers' problematization of patient participation indicates that they found this particularly challenging and points to the need to identify ways to support meaningful patient participation.
• The development of COS ideally needs to encompass the perspectives of stakeholders from countries in which COS are to be used.
• Methodological guidance that addresses the specific challenges developers encountered is a priority.
• Guidance on the application of methods to inform COS, such as systematic reviews, Delphis, and consensus meetings, was high priorities for developers, but the findings indicate that guidance on patient participation is important too.

## 1 Introduction

There are serious problems with selection and measurement of outcomes in clinical trials and health research. The outcomes used are not necessarily relevant to patients [1] or helpful in making decisions about health care. Inconsistency in which outcomes are measured is a major barrier to evidence synthesis and therefore to improving health care. The magnitude of inconsistency is striking. For example, over 25,000 of the outcomes in cancer trials have only been used once or twice [2]. Moreover, even when trials do measure the same outcomes, these are measured in such different ways that synthesis is often impossible. The extent of this problem is also striking: over 2,000 different measurement instruments have been used across 10,000 trials in schizophrenia, equating to a new instrument being introduced for every fifth trial [3]. A further problem is the incomplete publication of trial results, and particularly outcome reporting bias—the selective publication of a subset of the original recorded outcomes on the basis of the results [4], [5]. These problems lead to the use of ineffective, perhaps even harmful interventions, and to widespread waste of scarce health care resources [6].

A concerted effort is needed to address these problems. One solution is to develop agreed standardized sets of outcomes, known as core outcome sets (COSs). A COS is a list of outcomes that should be measured and reported, as a minimum, in all clinical trials in specific areas of health or health care [7]. If all trials in a particular clinical area used COS, the findings could be synthesized, and the resultant knowledge could be properly harnessed to benefit patients.

A systematic review of the 198 COSs published between 1981–2013 [8] indicated that a wide range of methods have been used to develop COS. Moreover, where similar methods have been used, these have been applied in different ways. While methods of development will likely need to be adapted to the context of a COS, variation in the methods used can influence which outcomes are ultimately included in a COS. COS development work by three different groups in the same clinical area (pediatric asthma [9], [10], [11]) that used different methodological approaches and involved different stakeholder groups resulted in some inconsistent outcomes being rated as important, although there was also some overlap in the outcomes prioritized across the three projects.

Most published COS have been developed in the absence of guidance about how to conduct COS studies. Indeed, the concept of a COS is still being established, and little is known about what should inform developers' methodological choices. As an emergent area of research, understanding developers' perspectives and rationales for their methodological choices will help to enhance future COS development. We therefore conducted qualitative interviews with COS developers about their experiences of COS development to understand the challenges involved, to inform methodological guidance, and to identify areas for future methodological research on COS development.

## 2 Study design and setting

Reflecting the aims of our study to inform practice, our approach was broadly pragmatic [12] yet interpretive, and we considered how participants constructed their experiences of COS development and what was latent in their accounts as well as the manifest content. The study received ethical approval from the University of Liverpool (reference: RETH000624).

It is important to outline the authors' interests in COS development, as these will inevitably have shaped the study and its findings. E.G. and P.R.W. helped to found the Core Outcome Measures in Effectiveness Trials (COMET) Initiative [7] in 2010. COMET promotes the development and application of COS and fosters methodological research to enhance COS. As members of the COMET Management Group, EG as Co-ordinator and PW as Chair, they have frequent contact with developers. They have also authored multiple COS publications, organized and participated in COMET conferences, and raised awareness about COMET worldwide. B.Y. also has interests in COMET, particularly stakeholder input to the development of COS and since 2015 has cochaired the COMET People and Patient Participation Involvement and Engagement Working Group.

### 2.1 Sampling

Sampling of COS studies was purposive for maximum variation [13], selecting published studies from a systematic review of COS [8] and ongoing studies from the COMET database (an online repository of COS). We operationalized the sampling via a matrix comprising: COS publication status (published vs. development ongoing); COS methods used; stakeholders included (patient input vs. no patient input); year of publication; COS scope (age of target group and intervention type); funding source (commercial vs. noncommercial), and a diversity of disease categories. In sampling published COS, we usually targeted those which had been published in 2010 or after to understand current COS development. Exceptionally, we interviewed developers of earlier COS when we anticipated that their accounts might be particularly informative, for example, their COS had been influential. For COS in ongoing development, we targeted those nearing completion as these developers' would have experienced more stages of COS development. For each of the sampled COS studies, we selected one interviewee only rather than interviewing multiple team members per COS, as this allowed us to sample a wider range of COS. We anticipated sampling 30–50 developers to reach data saturation, the point at which new data cease contributing to the analysis, and continued sampling until saturation had been reached.

### 2.2 Interviews

Semistructured, topic-guided interviews with developers explored aspects of COS development that we anticipated to be important, while also enabling interviewees to raise issues that were significant to them. Interviews were conversational involving a mix of open questions and more focused prompts. We informed participants that the interviews would explore their experiences of COS development and their methodological approaches. We did not send participants the topic guide before the interviews as we wished to avoid rehearsed responses. Development of the topic guide was informed by a systematic review of COS [8], by COMET Management Group members and the authors. Questions and prompts explored the methods developers had used, including their accounts of stakeholder input, methods of analysis, dissemination, and implementation (Appendix at www.jclinepi.com). The topic guide was developed iteratively over the course of the study, informed by the ongoing data analysis to explore previously unanticipated issues.

COS developers were geographically dispersed so were interviewed via telephone. There is no evidence that data quality is diminished by telephone interviews, when compared to face to face [14]. E.G. conducted all interviews in English. All interviewees gave signed, informed consent (by email) before interviews. Audio-recorded interviews were transcribed verbatim, checked and anonymized before being analyzed.

### 2.3 Analysis

**Fig. 1:**
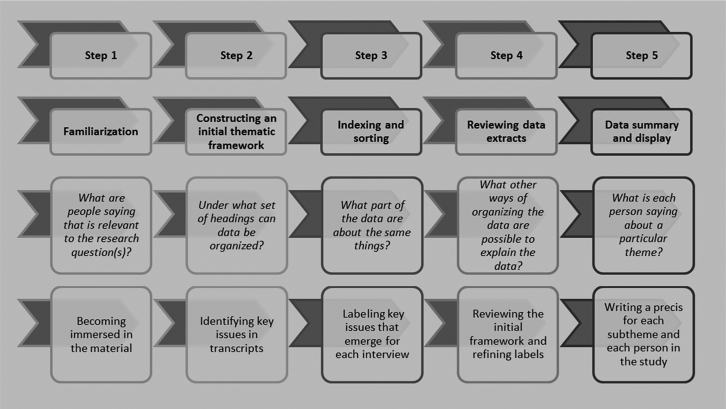
Five key steps of framework analysis.

Analysis drew on the constant comparative method [15], involving multiple and ongoing comparisons of data at different levels (i.e., from a fragment of text to entire transcripts) to explore and conceptualize the data [16]. E.G. led the analysis, which she periodically discussed with B.Y. and P.R.W., who read a sample of six transcripts and reviewed detailed reports of the developing analysis. We drew on framework analysis (illustrated in Fig. 1) to structure the analysis and maintain links between the data and developing findings [12].

Fig. 1 is based on the description of framework analysis by Ritchie et al. [12].

E.G. initially read the transcripts and checked summary field notes made immediately after interviews for additional details to inform her interpretations. Subsequent line-by-line analysis of transcripts informed the development of open codes. E.G. worked to develop the open codes into themes and categories by comparing data to identify recurring patterns and attending to outlier cases to help “test” and develop the analysis. Coding was supported by NVivo software [17]. E.G. developed framework matrices of themes to summarize the data, referring to field notes and whole transcripts as necessary for accuracy checking and to ensure themes were informed by the context of the full interviews. The matrices were further summarized through ongoing review and discussion with B.Y. and P.R.W.

The study and its reporting were informed by procedural guidance on quality in qualitative methods [18], [19], [20], although we note that such procedures cannot guarantee quality [21]. Therefore, we also judged the quality of the analysis by scrutinizing the insights it offered and the potential of these to inform practice in COS development [22], [23], [24].

The interview excerpts shown below were selected to explicate the findings and our interpretation of the data. Published developers are indicated by “P” and ongoing developers by “O”; “[…]” denotes text removed for brevity.

## 3 Results

### 3.1 COS study and developer characteristics

**Fig. 2:**
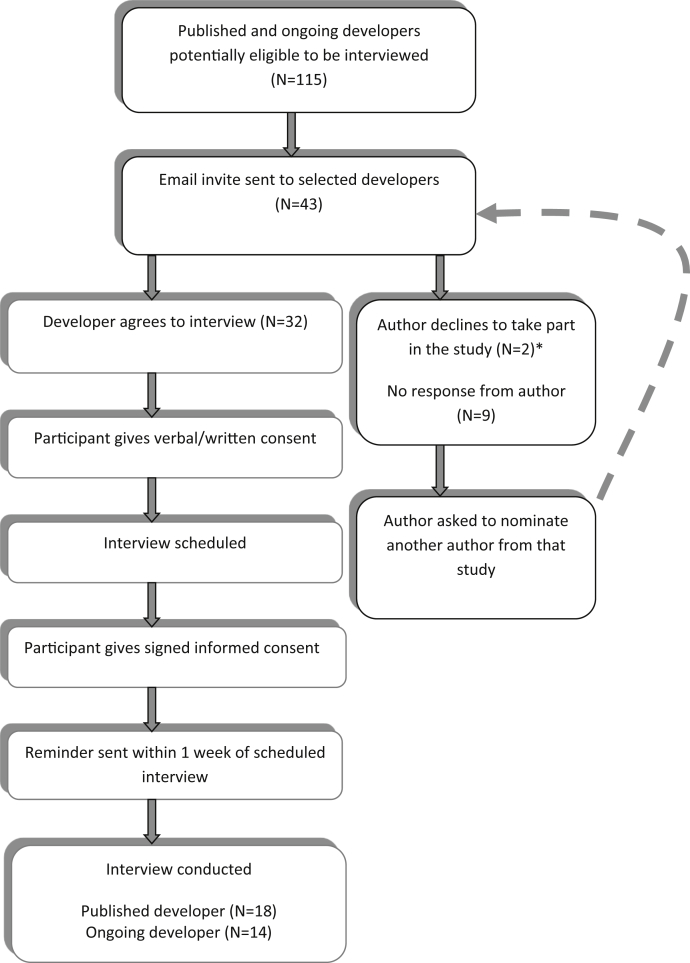
Participant selection and recruitment. *One participant did not give a reason for declining, and the other participant was “over committed.”

Fig. 2 summarizes the interview selection process. Of the 43 invited COS developers, 32 (74%) were interviewed between May 2014 and June 2015; 18 (56%) interviewees were based in Europe, 12 (38%) in North America, and 2 (6%) in Australasia. Eighteen (56%) had published COS, of whom 16 described themselves as lead authors, one as last and one as second author. Fourteen (44%) had ongoing COS projects, 10 of whom described themselves as principle investigators, 3 as the clinical leads, and 1 as the supervisor of a COS PhD project. None of the COS developers indicated they had backgrounds as patient research partners. Of the 32 COS discussed, 20 (62%) had participants from two or more countries, whereas 30 (94%) had clinical experts as participants and 20 (62%) had some input from patients. As we elaborate below, it was often difficult to discern whether patients had inputted as research participants or as research partners/team members. Further COS characteristics are summarized in Table 1.

**Table 1:** Summary characteristics of COS discussed in interviews

Characteristics	Published N = 18; number of COS (% of 18)	Ongoing N = 14; number of COS (% of 14)	Total N = 32; number of COS (% of 32)
Methods used (not mutually exclusive)			
Systematic/literature review	7 (39)	8 (57)	15 (47)
Delphi	7 (39)	10 (71)	17 (53)
Semistructured group discussion^a^	10 (55)	6 (43)	16 (50)
Consensus development conference	2 (11)	0 (0)	2 (6)
Nominal group technique	1 (5)	0 (0)	1 (3)
Focus groups	0 (0)	3 (21)	3 (9)
Survey	2 (11)	2 (14)	4 (12)
Unstructured group discussion^b^	1 (5)	0 (0)	1 (3)
Interviews	0 (0)	5 (36)	5 (16)
Methods not described	1 (5)	2 (14)^c^	3 (9)
Groups participating in COS development			
Clinical experts	16 (89)	14 (100)	30 (94)
Patients	8 (44)	12 (86)	20 (62)
Research methods experts	8 (44)	7 (50)	15 (47)
Representatives from regulatory authorities (e.g., FDA)	6 (33)	2 (14)	8 (25)
Representatives from industry (e.g., pharmaceutical companies)	5 (28)	1 (7)	6 (19)
Other (e.g., journal editors)	1 (5)	1 (7)	2 (6)
Not described	1 (5)	0 (0)	1 (3)
International participants			
Yes (participants from two or more countries)	11 (61)	9 (64)	20 (62)
No (participants from one country)	7 (39)	5 (36)	12 (37)
Study aims			
Outcomes only	11 (61)	12 (86)	23 (72)
Wider trial design issues	7 (39)	2 (14)^d^	9 (28)
How to measure			
What to measure only	8 (44)	12 (86)	20 (62)
What to measure + discussion/consideration of how but no recommendation	4 (22)	n/a	4 (12)
What + how (done together)	5 (28)	1 (7)	6 (19)
What + how (done in two stages)	1 (5)	1 (7)	2 (6)
Whether COS intended for adults or children			
Both	1 (5)	1 (7)	2 (6)
Children	4 (22)	3 (21)	7 (22)
Adults	3 (17)	10 (71)	13 (41)
Not specified	10 (55)	0 (0)	10 (31)
Type of intervention COS intended for			
All	3 (17)	8 (57)	11 (34)
Drug	4 (22)	0 (0)	4 (12)
Surgery	0 (0)	5 (36)	5 (16)
Specific (other)	3 (17)	1 (7)	4 (12)
Not specified	8 (44)	0 (0)	8 (25)
Number of existing COS/disease category			
Clinical area where more than 5	14 (78)	11 (79)	25 (78)
Clinical area where less than 5	4 (22)	3 (21)	7 (22)
Funding			
Commercial (e.g., drug company)	3 (17)	0 (0)	3 (9)
Noncommercial	7 (39)	13 (93)	20 (62)
Commercial and noncommercial	3 (17)	0 (0)	3 (9)
No funding	1 (5)	1 (7)	2 (6)
Not reported	4 (22)	0 (0)	4 (12)

*Abbreviation*: COS, core outcome set.

a Descriptions included workshop, meeting, and roundtable.

b Descriptions included task force, work group, working group/party, committee, board, and panel.

c Consensus methods, in addition to systematic review, were not known before the interview.

d Part of a wider PhD.

### 3.2 Overview

Developers spoke of COS development as a particularly challenging area of their work. In the sections that follow, we describe their accounts of these challenges, how it compromised their work, and the lessons they learnt. As we show, developers emphasized that the absence of guidance had affected their work and identified specific areas where guidance about how to develop COS would be useful.

### 3.3 The challenge of funding

Developers conveyed an overall sense of compromise in how they went about COS development arising from the difficulties of accessing funding for COS development and the limited funds available. As one developer explained, high expectations about how COS ideally should be developed contrasted markedly with the limited resources available:

The most difficult part […] was to combine the resources we had with what ideally should be done for developing a core outcome set. Because see, if you really want to do a systematic review of the outcomes included in clinical trials, if you want to do some qualitative research to have more the patient perspective […] and if you want to do a Delphi study like, as we did, so to focus also on both quantitative and qualitative perspective, it's, I mean, you know, quite some resources are needed […] it's not easy to get money in, in this field. So we had a limited amount of money, so we really had to match our resources with what we ideally wanted to do. (O31)

Developers were clear that the limited funds for COS development constrained their choice of methods, and in turn, compromised the quality of their work.

### 3.4 The challenges of stakeholder participation

Developers rarely distinguished between patient involvement in COS development, where patients contribute to the design of COS as research partners, and patient participation, where patients contribute data to COS development. However, as far as we could discern, COS developers who had included patients were referring to their inclusion as participants, and very few developers had included patients as research partners in their studies. Published developers who had not included patients anticipated that doing so would have been complicated and challenging and pointed to this as a reason for not including them in their studies. Several had published their COS before 2010 and remarked that it was somewhat unusual to seek the opinions of patients at that time.

Published and ongoing developers who had included patients remarked on the challenges that this brought. Many described how patients struggled to understand COS and to prioritize outcomes, or developers commented that patients rated everything as important, adding that research teams had struggled to convey to patients the need to limit COS to a feasible number of outcomes. One developer explained that the team had had to modify the question they asked participants from “which outcomes are important” to “what should be included into the core set” (P4). Having anticipated problems with patient participants' understanding of COS at the outset of their project, another developer emphasized the importance of anchoring questions to real interventions and “within the realm of possibility presently” (P5). This developer elaborated that when patients were asked what they would change about having their condition, they would answer “well I won't have [name of disease] anymore…that kind of almost magical thinking,” thereby attributing a lack of realism to patients about what is important to measure. No developers attributed a similar lack of realism to professional stakeholder groups such as clinicians or researchers.

In problematizing patient participation in COS development, developers tended to locate the difficulties with patients, rather than seeing the issue as one that the COS development community needed to resolve to enable patients to participate meaningfully. One of the few exceptions was a developer who spoke of the steps that developers could take to explain COS development to patients “in a way that will make them feel confident enough then to contribute” (P17). This developer emphasized the importance of ensuring that patients could recognize that “their voice was there” and detailed the need for appropriate phrasing and terminology for all stakeholder groups:

They're [patients] responding to this, we've got the right phrasing, and I think that's a really important aspect […] in that Delphi we took a lot of care around the phrasing so that the patients wouldn't be sort of alienated by the terminology used. And at the same time not alienating the professionals […] so there was sort of the more technical term that you might get in the literature alongside the wording that came from the qualitative data. (P17)

As noted above, no developers remarked that clinicians and researchers were unrealistic in their prioritization of outcomes and only a few developers described problems that had arisen in relation to professional stakeholders. One developer mentioned that clinicians found it “hard to discriminate between outcomes” (O20), while another described overcoming this challenge by asking participants to rank their top three outcomes instead of rating outcomes and singled this out as “one of the most important methodological choices” (P8) that the team had made. In their accounts of professional stakeholder participation, only two developers mentioned that clinicians or researchers' might lack understanding of COS, one remarking that if clinicians did not understand “they're going to guess and then that becomes an issue” (O32). This comment also indicates this developer's unease in working in an area that is fundamentally opinion based. The same developer added that clinicians might be reluctant to “admit” that they were uncertain of what was being required of them as participants in COS development because “doctors are not always very happy with saying, I don't know” (O32).

### 3.5 The challenges of research ethics approvals

When talking about the challenges of patient participation, some developers drew particular attention to requirements for research ethics approvals, and the time and money such processes demanded. They did not draw attention to these as barriers to the participation of other stakeholder groups, so underlining their problematization of patient participation. Indeed, some published COS developers gave the resource demands of ethics approvals as a reason for not including patients:

This was a one-year contract […] in order to involve patients in our process, we felt like to do it well we would want to get IRB approval to engage focus groups of patients, and felt like we didn't have sufficient time to do that unfortunately, or budget to sort of pay out incentives. (P6)

Elaborating on the resource intensiveness of research ethics procedures, which in the context of international COS development could require many different processes, another developer commented:

We wanted to do it with patients from different countries, but it was not easy […] in some countries we had to go through some ethical approval procedures that we were not prepared for […], so we were not able to involve patients from the UK for this reason, because we tried to do it through patients’ organisations in the UK but indeed we had to apply [for ethics approval] and we did not have time and resources […] so we did it only from other countries where there were not these ethical boundaries (O31)

In referring to how COS development work could be undertaken without research ethics approval, this developer points to a fundamental issue—whether COS development is actually research or not. Some developers elaborated on this, explicitly differentiating between including stakeholders as participants in research and “stakeholder work which is not classed as research” (O27). Referring to COS projects in the latter category, the same developer added: “when that happens you don't have the large workload of the ethics processes to deal with.” As well as raising questions about how developers are conceptualizing the status of COS development, whether as research or consultation, this implies that the practicalities surrounding ethics approvals rather than research principles are shaping how COS development is conceptualized.

### 3.6 The challenges of stakeholder sampling and retention

Developers described challenges in sampling and retaining the “right” participants. The challenges could arise in one or more stages of COS development including Delphi and consensus meetings:

Probably the biggest challenges are around trying to make the broadest outreach and assure that people who need to be in the room or need to know about will be invited to be in the room are there. (P13)

Developers therefore highlighted the importance of “who” is included as participants. One expressed concern about the difficulty of achieving diversity in patient participants and research partners for COS development and how not achieving this could lead to overlooking outcomes that were important for less advantaged patients (P17). This developer elaborated on the resource implications, noting that it takes more time to recruit a diverse group, and these participants may need extra support to allow them to participate.

Developers also described retaining stakeholders over the course of studies as a challenge:

The worry has always been lack of um engagement from clinicians, and secondly, even if you've engaged them for round one whether they'll stay on for round two is another matter. So there's a large imbalance in the two stakeholder groups at the moment, we've got about 110 patients for the patient group and we've got only 50 for the clinician group (O19).

Therefore as this developer noted, differential dropout between groups could give rise to imbalances between stakeholder groups.

### 3.7 The challenges of working internationally

A little over half of the developers had or were developing a COS on an international basis, which we defined as including participants from two or more countries (Table 1). In describing international COS development, developers emphasized the difficulties of accessing participants and funding, as well as the linguistic challenges that global participation entailed, including the need to translate concepts and questionnaires:

We kept our study within the UK, I would have liked to have done it more internationally but access to participants is an issue, and funding and various other things. We did our Delphi on paper so posting things internationally was going to be tricky, and then there's language issues and all this other stuff. (O23)

While developers often saw working internationally as desirable, resource constraints were a limiting factor and COS developers engaged in a trade-off between what would be ideal and what was pragmatic:

It's best if these things are international actually, but I think we have to think very carefully about what we are doing the research for and whose care we might be improving. We also need to think very carefully about the resources we've got available and that includes financial resources […] but it might be that sometimes we just have to do work in a single country […]. Sometimes that's a pragmatic approach to take. (O27)

Noting the need for research on two contrasting approaches to international COS development, another developer explicitly linked questions about the feasibility of working internationally to the overall goal and scope of the COS in question:

I don't know if the step would be to have all stakeholders, you know, from all different countries in the same room, or whether there should be parallel processes in different countries that potentially reach very different conclusions but that should be sort of compared […] but […] the goal was not to sort of get international perspective, it was to get a US perspective. (P6)

Developers were therefore aware that developing a COS for international use has several implications, including for the sampling of participants and for the resources required.

### 3.8 The challenges of publication

Preparing protocols describing the development process is advocated as a way of addressing the potential for bias in COS development [25], aiding transparency and assisting methodological development. However, ongoing developers described challenges in publishing their COS protocols, with one even referring to the process as “painful” (O19). In all instances, developers linked these challenges to reviewers' and editors' perceived lack of understanding or knowledge about COS, the development process, and the importance of COS:

It wasn't really easy to publish it […] not many people are actually experienced, are experienced in core outcomes, and they are not very experienced in […] the e-Delphi […] and from their [the journal] reviewer point of view you would get you know very funny sort of questions and critiques, which, which just wouldn't make sense. (O30)

When it came to publishing completed COS studies, several developers anticipated difficulties at this stage, but only one published developer described having actually experienced problems when publishing COS study findings. This developer spoke of the “disparaging” comments he had received from a couple of high impact journals, which included “this is a nice idea but is it really science?” (P8). This echoes earlier questions about the status of COS development as research. Developers also expressed concerns that the time required to develop COS might mean that the COS was obsolete when finally published, as “new developments, new interventions come into the equation” (O19).

### 3.9 The challenges of implementation

Developers pointed to the importance of ensuring the uptake and implementation of COS:

You can have the best methodology in developing a core outcome set but if at the end of the day the, no one uses it, you cannot really say that your, the development of your core outcome set was successful. Implementation is not an easy step but it is really worth it to spend time and energy on that. (031)

While this particular comment was made by an ongoing developer, published developers tended to show more awareness of the challenges of ensuring the uptake and implementation of the COS than ongoing developers, with some published COS developers describing it as the “biggest challenge.” This is perhaps unsurprising, as published developers are likely to have more experience of the challenges associated with implementation than ongoing developers, whose focus seemed to be on more immediate concerns of development. Indeed, some ongoing developers seemed to be deferring their efforts to promote implementation:

I don't see it [implementation] too much as a challenge now […] but this might be a challenge after some years when our core outcome set will be developed and we will be seeing maybe that people don't use it too much. (031)

Other COS developers described instances where COS implementation had been unsuccessful and they did not know how to resolve the situation. For example, a few developers described a lack of openness by gatekeepers to the uptake of their COS:

Right now, studies, are being planned and conducted on systemic treatments for [name of disease] and the FDA [U.S. Food and Drug Administration] just doesn't accept our core outcome set. They favour an outcome that is not included in the core outcome as a primary endpoint, and they just refuse to acknowledge our work. And we wrote them, I think four or five months ago, about this issue and we never got a reply. (P4)

Developers were aware that the process of COS development could influence its implementation. Describing this as “social and political” aspects of COS development, one COS developer saw a close link between who is involved in the development of a COS and the success of its subsequent uptake:

Maybe people who are not directly involved in the development of a core outcome set are not very happy to […] use this core outcome set, certainly people, many people agree that standardisation is important […] but many people do not want to use standardisation developed by others. (O31)

In speaking about the barriers to implementation of COS, ongoing and published developers emphasized the research community's lack of understanding of the purpose and importance of COS. Interestingly, this contrasts with their accounts of stakeholder participation in COS development, in which they gave relatively little emphasis to the problems that professional stakeholders might have in understanding COS.

### 3.10 The need for COS development guidance and how specific it should be

Developers described the need for guidance about how to develop a COS. They commented on a lack of understanding among researchers generally of the process to develop COS “correctly,” and of doing the best they could when developing their COS in the absence of guidelines:

There is a bit of a lack of, I don't know, understanding of, you know core outcomes process, and why it's actually needed, and you know, how it should be done correctly. I am not saying that what I've added was 100% correct in everything, but […] you can do as much as you can do if there are no guidelines. (030)

The potential for guidance to assist in the dissemination and uptake of COS was particularly prominent in developers' accounts. For example, again referring to a lack of understanding about COS in the research community, “a lot of journals don't necessarily know about this methodology yet either” (O22), one developer anticipated that guidelines about methods would assist in publishing COS as developers could refer to guidelines to justify their methodological decisions. Published developers tended to speak of a “best” methodology, implying that there could be one “right” method for COS development or referred to guidance which “prescribed” “a concrete set of steps to go through” (P2). Indeed, P2 saw considerable merit in guidelines that were highly specified as a way of distinguishing quality and lending credibility to COS:

People could say wow they really did not stick to what is considered a robust methodology for creating a core outcome set, I really have no faith in the final product or the converse, they did everything they could given the resources that they had and I do think that future investigators should lean on that paper. Or not. (P2)

Ongoing developers also felt that guidance was needed, but unlike published developers, they tended to acknowledge that “a one size fits all approach” was untenable due to the “nature” of COS development (O19). O19 was one of several developers who articulated that guidance should help developers “think for themselves” and noted how the methods that developers used would need to be adapted to the disease context or circumstances in which developers were working.

#### 3.10.1 The content of guidance: what developers wanted

All developers wanted guidance on COS development, but it was mostly ongoing developers rather than published developers who identified the specific content and methodological areas where guidance would be helpful. Referring to her work as preceding the “methodology movement” (P18), one published developer hinted that COS developers were becoming more methodologically aware. The specific areas of COS development that developers identified as priorities for guidance or research are listed in Table 2.

**Table 2:** Areas of COS development where developers identified that guidance or research was needed

Area of COS development	Illustrative quotes
***Systematic review of outcomes***	
Domain categorization^a^	The domain categorisation I think is something which needs to be highlighted. I think you need to be really careful about how you do it. It is ultimately quite a subjective process, and I think you need to have methods in place that mean that, you have appropriate categorisation […] if you don't get that stage right then your Delphi just kind of doesn't matter. (023)
***Delphi***	
Response rates	The biggest challenge that we faced was trying to get enough responses to the online survey. Despite [name of charity organisation] help, we struggled to get as many responses as we would have liked and we went to, we looked at the variety of ways around that including advertisements in the press and other things. (O24)
Feedback	Within Delphi, I don't think anyone understands the best type of feedback to give people, the impact of what that feedback, you know of how that feedback affects responses. […] Do you combine different stakeholders, different results at different stages, do you merge it all at the end? Yeah these are all sort of mysteries to be honest with you. I could go on and on. (O23)
Balancing stakeholder groups^b^	So there's a large imbalance in the two stakeholder groups at the moment, we've got about 110 patients for the patient group and we've got only 50 for the clinician group. (O19)
Retention of outcomes between rounds	The issues around whether you discard or retain outcomes or whether you keep the whole lot in until the end of the Delphi process, I don't think that's been resolved at all. (O23)
Sampling	How do you know, and how can you demonstrate if you do know, that the people you've chosen are the right people? (O28)
Sample size	I certainly think in terms of guidance, I think numbers of people for a Delphi, some information about that would be useful 'cause there really was nothing about that at all. (O29)
Bias	How do you motivate people to answer? If you're motivating people to answer them, is that not biased? (O32)
***Consensus meeting***	
Conduct and management	Over the, I guess, 20 years or so that I've been involved in chairing discussion meetings, I've learnt on the job, and I don't think that's necessarily the best way […] it's good to be able to benefit from other people's experience. But I didn't really come across guidance on how to run discussion meetings that I found useful. (P14)
Organization and efficiency	Guidance should include how best to organise a workshop like this to try and ensure that you use the expertise and time of the individuals actually face to face as efficiently as possible. (P14)
Objectives	The functionality of the consensus meeting is very, very vague. In the, in the literature, there is absolutely no consensus there about […] what are the objectives and the aims of having this consensus meeting, so that is something that needs to be looked at. (O26)
***Reporting***	
Reporting findings	How to report your findings, 'cause I think that it’s only very few papers that actually highlight, you know, best practice in reporting findings for the consensus. […] And especially when you compare it to previously done, previously published papers, you see that it sort of quite a few of the papers are missing very important points. (O26)
***Patient participation and involvement***	
Number of patients and meaningfulness of patient participation	I certainly think in terms of guidance […] number of patients as well, but […] for every clinical context that will probably differ and the amount of involvement that you have from patients will, you know, differ depending on the clinical context. (O29)
Resources	It's the same with PPI – patient involvement in research – a lot of groups won't have the resources to do that properly. So I think one has to be careful that um any kind of description of what gold standard is, is also tempered by a, a reality check about what's do-able within small resource envelopes. (O27)
Sampling and recruitment	How to identify stakeholders, it's hard to. Well for healthcare professionals […] you can more easily I guess identify people that are working on that field […] for parents it's much harder […] You might use a social media strategy […] but [if] you identify parents that have had [name of disease] at a national level, now does that add much in terms um the methods as compared to a parent workshop that is adequately sampled based on severity of the condition or a level of care? (O20)
Diversity	It worries me a lot that the people who are being involved in this way on a sort of patient research partner level, but probably as participants generally, um don't represent that diversity, and so we're missing potentially priorities, you know, a different set of priorities. (P17)

a Outcome domains are constructs used to classify similar outcomes or group broad aspects of the effects of interventions (e.g., functional status) into categories.

b Delphis are used to elicit group opinion and reach consensus, using the rationale that “n heads are better than one” [26]. Opinions of individual participants are surveyed over several rounds and asked to review and rescore items based on feedback on aggregated scores from previous rounds. Different participant subgroups might have different sample sizes.

## 4 Discussion

### 4.1 Summary of findings

This study indicates that COS developers struggled with many challenges and uncertainties over the course of their projects. Although developers pointed to the lessons, they had learnt throughout their studies, as an emerging field without shared assumptions or an advanced methodological knowledge base, it was clear that COS development would benefit from methodological guidance.

We were particularly struck by how developers problematized patient participation, in comparison to the participation of other stakeholders. Developers tended to locate the problems with patients rather than as a challenge for the COS development community to resolve by developing better explanations for patients and better ways of seeking their input. By asking patients to participate in COS development, in effect, developers are asking patients to enter into the world of research [27] and appreciate, for example, that only a relatively small number of outcomes can be measured in research. It follows that it is the responsibility of the COS development community to identify ways to support meaningful patient participation in COS development, and take care to facilitate the insights patients can provide and avoid seeing their input as somehow lacking when it challenges the perspectives of researchers or clinicians. Involving patients as research partners or team members in COS development could help COS teams better support patient participants [27]. The COMET website provides resources to help developers facilitate patient participation, including documents explaining COS development in plain English (http://www.comet-initiative.org/resources/PlainLanguageSummary).

The dividing line between “participation in research” and “involvement in consultation” is arguably not so clear in COS development compared to other areas of research, as participants in a Delphi exercise or consensus meeting are in effect being asked their opinions about a research design issue. Developers' accounts of research and consultation became particularly blurred when speaking about ethics requirements. Some had doubts about when ethical approval was needed for seeking patient input to COS. Others saw classifying patient input to COS as consultation rather than research as a way of obviating the need for research ethics approval, with attendant time and cost savings. However, at least in the UK and United States, the Health Research Authority (http://www.hra-decisiontools.org.uk/ethics/) and Institutional Review Board (http://www.irb.umn.edu/research.html) are clear that where the purpose of an activity is to produce generalizable knowledge, the activity is research rather than consultation, and ethics review is required. Ultimately, COS development aims for generalizable knowledge, and if COS is to have the status of research, ethics review will be required for all participants, not just patients.

Uncertainties about the status of COS have wider implications, particularly for the implementation of COS, as indicated by developers' references to “social and political” aspects of the process and the importance of including a diversity of stakeholders. A related issue was apparent in the unease that some developers had about the opinion-based nature of COS development and a developer's account of influential journals questioning whether COS development is “really science.” Such questions also warrant further consideration in developing support and guidance for COS. For example, many COS developers and reviewers are likely to have trained in the natural sciences and may not be aware that opinions (though subjective) can be investigated in disciplined and systematic ways. Paradoxically, while developers' were often aware of the importance of methodological principles and the need for COS to be generalizable, in describing their direct experience of the process, it was practicalities, such as available funding or access to stakeholders, rather than principles, which influenced their choices and contributed to a blurring of the distinction between research and consultation. Clearly, guidance is needed on the fundamental issue of whether COS development is research and linked to this, what ethics approvals are required.

Questions about resources and practicalities were particularly prominent in developers' accounts of international COS development. Additionally, developers had concerns about the heterogeneity of views that might arise when participants are included from multiple countries, or conversely, about the broader generalizability of COS that were developed in one country. Developers pointed to the challenges of working across multiple languages and to the resources required for translation. While techniques for translation in other research contexts are available [28], [29], implementing these in COS development, where Delphi questionnaires will inevitably vary from one COS to the next, is likely to be prohibitively expensive. This is another area where guidance for developers would be helpful, as well as further research to explore the feasibility of different models of international COS development.

Developers clearly wanted guidance on the process of COS development, but published and ongoing developers differed in their views about how prescriptive such guidance should be. We would argue that highly prescriptive guidance might inhibit development by not allowing developers the flexibility to tailor their methods to contextual issues or level of resourcing and therefore might even undermine quality in COS development. Developers also commented that guidance on how to report COS work would aid future COS developers. Such a reporting guide has recently been published [30], while work is underway to develop minimum standards for conducting COS studies.

In contrast to published COS developers, ongoing COS developers gave detailed accounts of the methodological uncertainties that had perplexed them. This might reflect the recency of their experience or it could indicate a greater awareness among ongoing developers of the complexities of COS development, through the work of groups such as the COMET Initiative [8], [31] and OMERACT [32], [33]. Ongoing developers particularly prioritized the need for guidance on how to do systematic reviews within the particular context of COS development, as well as how to conduct of Delphis and consensus meetings. As these are among most frequently used methods in COS development, there is an impetus for research to answer questions about best practices in using these methods in COS studies. COMET have produced a Handbook identifying accumulating methodological work in this area, informed by this study, and other research [25]. This Handbook will be updated as new evidence becomes available.

Implementation of COS also posed challenges for developers. Their accounts indicated that the success of implementation will not just be a product of the quality of the methods used and that efforts are needed to include the “right” participants and ensure that the relevant communities have ownership of the COS. Such efforts will likely be more fruitful if developers get relevant stakeholders on board and raise awareness of their work from the start of their projects. It is therefore critical that future guidelines emphasize the importance of working toward successful implementation from an early stage in the COS development process. COMET has been active in promoting the implementation of COS and UK and European research funders such as National Institute for Health Research (Health Technology Assessment programme), Arthritis Research UK, Horizon 2020, and the Irish Health Research Board now encourage applicants to consider COS when seeking funding for new trials. Furthermore, a recent guideline on pediatric asthma trials recommends the use of COS [34]. We suggest that alongside COMET, developers have a critical role in raising awareness and gaining interest in COS within their clinical and research communities.

### 4.2 Strengths and weaknesses of the study

This study has provided insights into COS development from the perspective of developers. By purposively sampling across a range of COS that encompassed a variety of development methods, we anticipate that our findings will be broadly transferable. Moreover, by including COS in ongoing development as well as published COS, the areas identified as priorities for guidance are likely to remain current for informing practice in COS development for a number of years.

This study describes the experiences of developers who agreed to be interviewed. Furthermore, while we identified published developers via an extensive systematic review of published COS, we identified ongoing developers through the COMET database, which comprised only those who had registered with COMET. Therefore, we do not know the extent to which our findings will be relevant to ongoing developers whose work is not registered with COMET.

## 5 Summary

This study aimed to describe experiences of COS development and to identify priority areas for future guidance and methodological research. Developers found COS development to be a challenging process, likely in part due to its relatively recent emergence as a field of research. The findings raise important questions about the status, funding, and process of COS development and highlight areas that future research should focus on to strengthen COS development. Guidance needs to simultaneously highlight common issues, encourage COS developers to think about their own contexts and circumstances, as well as enable them to make decisions about methods that best suit their needs and resources.
