# Supplementary material for: Improving core outcome set development: qualitative interviews with developers provided pointers to inform guidance
Source: J Clin Epidemiol. 2017 Jun;86:140–52. doi: 10.1016/j.jclinepi.2017.04.024 (PMC5513443; doi:10.1016/j.jclinepi.2017.04.024)
Supplement: Appendix [file mmc1.docx]

**Appendices**

Appendix 1: Version 1.0 of the interview topic guide for interviewing COS developers

**Background**

- Tell me about your role in the development of this core outcome set
- Tell me about your research [title]
- How did this study come about?
- What role did each individual have in the planning of this work

**Choice of methods**

- Can you talk me through the planning of this project
- Was the study funded?
- Can you tell me about the methods you used?
- Do you think that the available resources influenced the methods you used?
- Did the methods change throughout the process?
- Did you experience any difficulties in maintaining interest in the process?

**Choice of stakeholders to include**

- Who was included in the development of this core outcome set (*as participants not as members of research team)*?
- How did you decide who to include in this work?
- How did you decide how many and the proportions of different types of people to include?
- Did you end up including the numbers and proportions you intended?
- How did you explain the study to different groups?
- Did you think about including any groups that you didn’t end up including?
- How did you decide to integrate opinions from different stakeholders?

**Process and methods of analysis**

- How did you decide what information (if any) to give to participants before the process?
- How did you decide on how outcomes would be scored or rated during the consensus exercise or at each stage in the process?
- Did you adopt a particular definition for consensus?
- How did you decide the procedure for determining how outcomes would be included or excluded from consideration at each stage of the consensus process?
- How did the researchers ask the question(s) about outcomes?

**Results**

- What did you think about the content of the core outcome set at the end of the study?
- What influenced the final choice of outcomes to include in the final core outcome set?
- Were there differences in the outcomes identified and thought to be important by different groups?
- Did the development of this core outcome set change anything?
- Did the results influence your thinking?

**General**

- Can you tell me about the publication process for this project
- What (if any) have you experienced to be the challenges of this work?
- What (if any) have you experienced to be the benefits of this work?
- Would you do anything differently?
- Were there any areas particularly that you felt would benefit from methodological research?
- Did you go as far as to consider the implementation of your core outcome set?
- Do you think there were any limitations to your work?
- What do you think are the implications for future research?
- What impact do you think this work will have?
- What is it intended the core outcome set is used for?
- Do you think there were any conflicts of interest within the study team?
- Do you plan to work on another core outcome set?
